# Compositional Effects of the Structure and Properties of 3D Printed Stratified rPET/rPETG Shape Memory Composites

**DOI:** 10.3390/polym18030370

**Published:** 2026-01-30

**Authors:** Ștefan Dumitru Sava, Vasile Ermolai, Bogdan Pricop, Radu-Ioachim Comăneci, Corneliu Munteanu, Nicoleta-Monica Lohan, Mihai Axinte, Leandru-Gheorghe Bujoreanu

**Affiliations:** 1 Faculty of Materials Science, “Gheorghe Asachi” Technical University of Iași, Blvd. Dimitrie Mangeron 71A, 700050 Iasi, Romania; stefan-dumitru.sava@student.tuiasi.ro (Ș.D.S.); bogdan.pricop@academic.tuiasi.ro (B.P.); radu-ioachim.comaneci@academic.tuiasi.ro (R.-I.C.); nicoleta-monica.lohan@academic.tuiasi.ro (N.-M.L.); mihai.axinte@academic.tuiasi.ro (M.A.); 2 Faculty of Machine Manufacturing and Industrial Management, “Gheorghe Asachi” Technical University of Iași, Blvd. Dimitrie Mangeron 59A, 700050 Iasi, Romania; vasile.ermolai@academic.tuiasi.ro; 3 Faculty of Mechanical Engineering, “Gheorghe Asachi” Technical University of Iași, Blvd. Dimitrie Mangeron 61-63, 700050 Iasi, Romania; corneliu.munteanu@academic.tuiasi.ro

**Keywords:** shape memory effect, glass transition, stratified composite, recycled PET, recycled PETG, 3D printing, thermomechanical cycling, storage modulus, tensile testing, fractographs

## Abstract

The paper continues the authors’ efforts to characterize and control the shape memory effect (SME) occurring in 3D printed specimens of recycled polyethylene terephthalate (rPET) and polyethylene terephthalate glycol (rPETG). Lamellar and “dog-bone” configuration specimens were 3D printed in the form of stratified composites with five different rPET/rPETG ratios, 100:0, 60:40, 50:50, 40:60, and 0:100, and two different angles between the specimen’s axis and the deposition direction, 0° and 45°. The lamellar specimens were used for: (i) free-recovery SME-investigating experiments, which monitored the variation of the displacement, of the free end of specimens which were bent at room temperature (RT), vs. temperature, during heating, (ii) differential scanning calorimetry (DSC), which emphasized heat flow variation vs. temperature, during glass transition and (iii) dynamic mechanical analysis (DMA), which recorded storage modulus vs. temperature in the glass transition interval. Dog-bone specimens were subjected to tensile failure and loading-unloading tests, performed at RT. The broken gauges were metallized with an Au layer and analyzed by scanning electron microscopy (SEM). The results showed that the specimens printed with 0° raster developed larger free-recovery SME strokes, the largest one corresponding to the specimen with rPET/rPETG = 40:60, which experienced the highest storage modulus increase, 872 MPa, and maximum value, 1818 MPa, during heating. The straight lamellar composite specimens experienced a supplementary shape recovery when bent at RT and heated, in such a way that their upper surface became concave, at the end of heating. Most of the specimens 3D printed at 0° raster developed stress failure plateaus, which were associated with the formation of delamination areas on SEM fractographs, while the specimens printed with 45° raster angle experienced necking failures, associated with the formation of crazing areas. The results suggested that 3D printed stratified rPET-rPETG composites, with dedicated spatial configurations, have the potential to serve as executive elements of light actuators for low-temperature operation.

## 1. Introduction

Polyethylene terephthalate (PET) and polyethylene terephthalate glycol (PETG) are two related polymers commonly used in daily life. Despite being related, their global production figures are quite different. The former has exceeded 80 Mt/year, being ranked in fourth position, with 6.2% of global plastics production in 2022 [1], while the latter was estimated at only tens of kt/year [2].

Considering these huge production figures, both polymers have inevitably contributed to the production and accumulation of large amounts of plastic waste. Consequently, sustained efforts have been made throughout the world to manage their recycling. In this way, the recycled forms of these polymers have emerged as rPET [3] and rPETG [4]. One of the most effective ways to leverage the applicative potential of rPET and rPETG is to utilize them for 3D printing in either filament or granular form [5]. For this purpose, a better control of the properties of 3D printed items from recycled thermoplastics was achieved [6]. This was accomplished by the optimization of 3D printer settings [7], which significantly enhanced the mechanical properties of both filament and 3D printed rPET parts [8]. In addition, both at filaments and 3D printed specimens, the occurrence of shape memory effect (SME) was reported, since they recovered their undeformed permanent shapes, during heating beyond the temperature of glass transition, after being deformed to a temporary shape at room temperature (RT) [9].

In three previous articles, some of the present authors have reported the presence of free-recovery SME at rPET [10], the occurrence of both free-recovery and work-generating SME at rPETG [11], and an increase of both static and dynamic stiffness at the end of a heating-cooling cycle at rPETG [12], respectively.

The comparative studies of the stress-strain behaviors of rPET and rPETG revealed differences mostly in plane strain deformation [13], emphasizing the superior mechanical properties of the latter as compared to the former. In addition, rPETG featured the capacity to develop free-recovery SME for up to 20 consecutive bending cycles and a work-generating SME exceeding 0.5 J/kg [11].

These previous results suggested that the properties of rPET and rPETG could be combined by 3D printing of multi-material parts based on the face-to-face bonding interface between the two thermoplastics [14], such as stratified mixtures between rPET and rPETG layers [15]. For this purpose, 3D printed rPETG/rPET composites will be analyzed as a solution for the recycling of scraped plastic [16].

More concretely, stratified composites with various rPET/rPETG ratios and two different layer deposition directions will be manufactured by 3D printing and analyzed from the point of view of the compositional effects on their structure and properties. Using recycled materials for shape memory applications is meant to contribute to a more efficient management of plastic waste.

## 2. Materials and Methods

### 2.1. Specimens Preparation

The raw materials for 3D printing were black rPET filaments produced by FormFutura (Nijmegen, The Netherlands) and rPETG filaments manufactured by GreenTech SA Company (Buzău, Romania).

Before printing, the filaments were dehydrated on a Sunlu FilaDryer S4 device (Kowloon, Hong Kong) at a temperature of 50 °C for 24 h, achieving a relative humidity of 14%.

**Figure 1:**
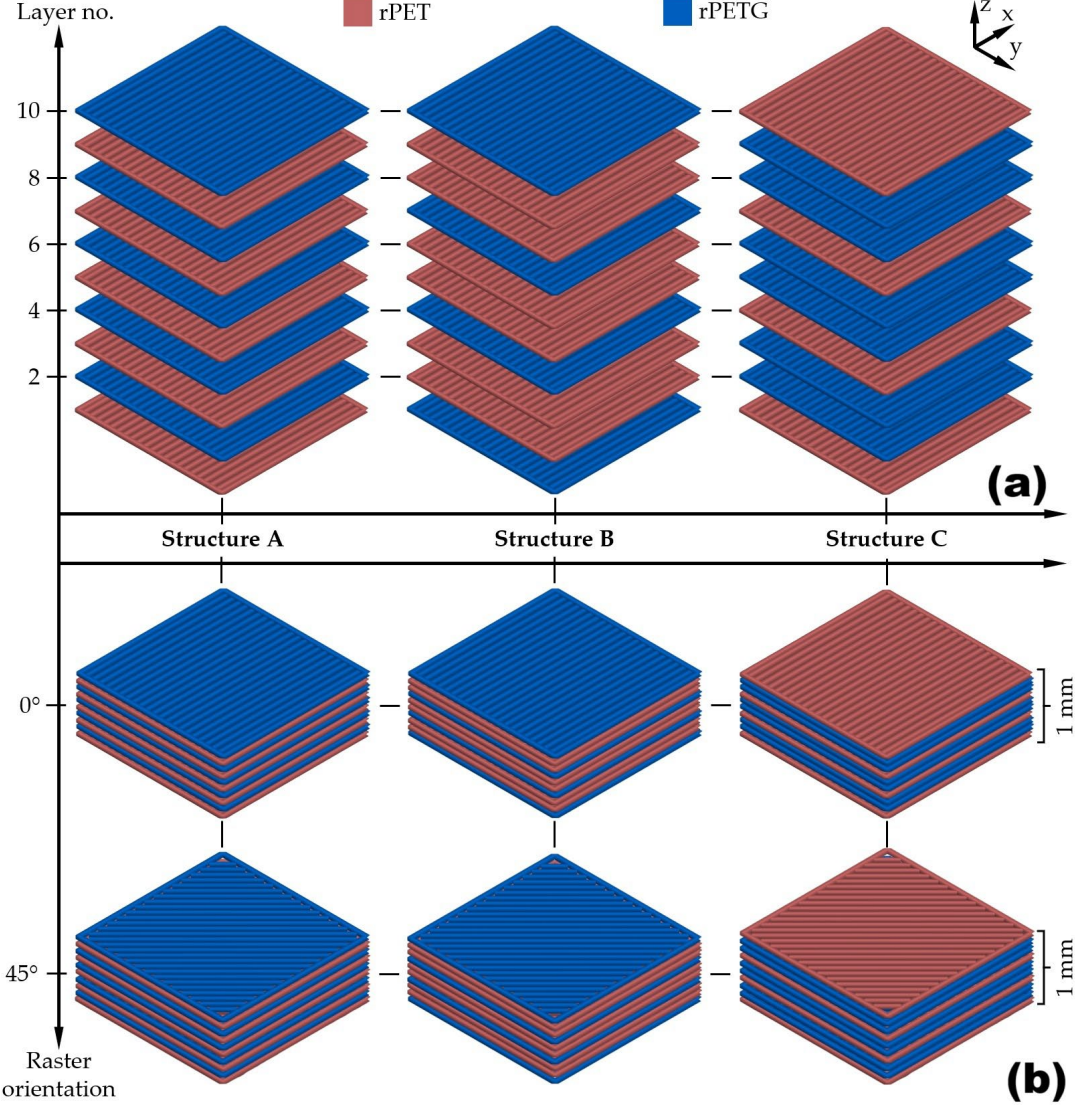
Printing schemes of stratified composites. (**a**) layer succession for the specimens A-50/50 rPET/rPETG, B-60/40 rPET/rPETG and C-40/60 rPET/rPETG; (**b**) emphasizing raster orientation.

During 3D printing, the filaments were stored in a PolyBox recipient produced by Polymaker (Houston, TX, USA) with silica gel, meant to keep the relative humidity below 15%. The 3D printing was performed on a printer fabricated by BambuLab A1 company (Shenzhen, Guangdong, China), using a 0.4 mm nozzle without enclosure, with an AMS system for filament automatic changing under laboratory conditions, at a temperature of 26.1 °C and a humidity of 51%. The process parametrization was accomplished with OrcaSlicer 2.3.0 software (SoftFever, San Antonio, TX, USA). The 3D printing parameters and schemes for three types of composite specimens are listed in Table 1 and Figure 1, respectively.

As noticeable from Figure 1b, two deposition directions of the filament were used: 0° and 45° to investigate the effect of raster orientation on the structure and properties of stratified composites.

In addition to stratified composites, two types of specimens were 3D printed as 100% rPET and 100% rPETG. These two types of specimens will be further designated as rPET and rPETG, respectively. The three rPET/rPETG proportions were selected from the point of view of the optimum 3D printing succession of the stratified composite. The “central” proportion was rPET/rPETG = 50:50, which implied a simple succession of single different polymer layers, as in structure A, from Figure 1. The other two proportions, 40:60 and 60:40, were obtained by printing a succession of 1-2-1-2 … layers from different polymers, according to the structures B and C from Figure 1. Increasing the number of layers printed with the same polymer (e.g., 1-3-1-3 …) could compromise the homogeneity of the tensile response of the stratified composite [17] In conclusion, ten types of specimens were printed, differentiated by the rPET/rPETG weight percentage ratio and raster orientation: (i) rPET-0 (100 rPET/0 rPETG printed at 0°); (ii) rPET-45 (100 rPET/0 rPETG printed at 45°); (iii) B-0 (60 rPET/40 rPETG printed at 0°); (iv) B-45 (60 rPET/40 rPETG printed at 45°); (v) A-0 (50 rPET/50 rPETG printed at 0°); (vi) A-45 (50 rPET/50 rPETG printed at 45°); (vii) C-0 (40 rPET/60 rPETG printed at 0°); (viii) C-45 (40 rPET/60 rPETG printed at 45°); (ix) rPETG (0 rPET/100 rPETG printed at 0°) and (x) rPETG (0 rPET/100 rPETG printed at 45°).

Using the above 3D printing technique, specimens with lamellar and “dog-bone” configurations were manufactured for each rPET/rPETG ratio and raster orientation.

The lamellar specimens had the dimensions 1 × 4 × 50 mm, and the “dog-bone” ones were fabricated according to ISO 527-2, a standard which was also used for the calculation of Young’s modulus for a yield strain of 0.5% [18].

### 2.2. Experimental Methods

The occurrence of free-recovery SME in bending was investigated according to the previously detailed procedure, which involves the following stages: (i) fastening the lamellar specimens at one end; (ii) bending them to 90° at RT, and (iii) heating with a hot air gun to 100 °C [7,8]. By cinematographic analysis, the variation in the vertical positions of the specimen’s free end, during heating, was monitored and examined frame-by-frame [19], with measuring precisions of ±1 mm for the vertical displacement and ±2 °C for temperature.

Differential scanning calorimetry (DSC) analysis was performed by means of a NETZSCH DSC 200 F3 Maia device (Netzsch, Selb, Germany) calibrated with Bi, In, Sn, Zn, and Hg standards. For experiments, parallelipipedal fragments weighing between 10 and 16 mg were cut from lamellar specimens, which were heated up to 100 °C, with a rate of 10 °C/min, in order to emphasize the occurrence of glass transition, associated with an endothermic step of heat flow variation with temperature [20]. The experiments were carried out in an inert gas atmosphere, using Ar. The specimens were placed into Al crucibles with inner diameters of 5 mm and a depth of 2 mm, which were covered by Al lids.

The specimens’ stiffness variation during heating was monitored by dynamic-mechanical analysis (DMA), using a NETZSCH DMA 242 Artemis device (Netzsch, Selb, Germany) equipped with a dual cantilever specimen holder, according to the previously detailed procedure [10]. Lamellar specimens, with the dimensions 1 × 4 × 50 mm, were dynamically bent, with an amplitude of 100 µm and a frequency of 1 Hz, while being heated from RT up to 90 °C with 5 °C/min. Storage modulus variations with temperature were recorded to determine the temperature range where a marked softening occurred, during heating, which is typically associated with glass transition [21].

The evaluation of both DSC and DMA thermograms was performed using the Proteus software, v.6.1, Selb, Germany.

The static tensile behavior of the specimens of rPET/rPETG stratified composites was evaluated by RT failure and loading-unloading tests performed on an INSTRON 3382 tensile testing machine with thermal chamber (Norwood, MA, USA), with a cross-head speed of 1 mm/min. The machine has a maximum load of 100 kN, a maximum speed of 508 mm/min, a total vertical test space of 1430 mm, and the reading down sensitivities of ±0.5% to 1/200 of the load cell capacity and ±1% from 1/200 to 1/500 of the load cell capacity. The tensile strain was measured with an INSTRON 2620 clip-on extensometer with a gauge length of 25 mm and a linearity of 0.15/full scale. After the failure tests, the fractured surfaces of “dog-bone” specimens were metalized with a 10 nm-thick gold layer, which was deposited by means of a LUXOR Au/Pt Coater (APTCO, Berlin, Germany). The metallized specimens were analyzed by scanning electron microscopy (SEM), using a VEGA II LSH TESCAN device (TESCAN, Brno, Kohoutovice, Czech Republic).

## 3. Results

### 3.1. Evaluation of Free-Recovery SME

**Figure 2:**
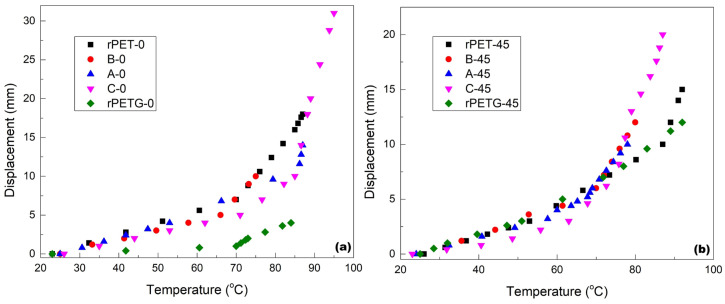
Variations of the free-end displacement vs. temperature of the rPET/rPETG lamellar stratified composite specimens, 3D printed with different raster orientations: (**a**) 0° and (**b**) 45°.

The occurrence of free-recovery SME in the lamellar specimens of rPET/rPETG lamellar stratified composites is illustrated in the videos from the S1 Supplemental Material. By determining the position of the specimen’s free end at different temperatures, at every 2 s, the diagrams illustrated in Figure 2 were obtained.

By comparing the two diagrams, the following particularities can be noticed:

(i) Most of the specimens 3D printed at 0° developed larger strokes as compared to those printed at 45°;
(ii) The specimens C (rPET/rPETG = 40/60) and rPET developed the largest strokes and
(iii) The maximum stroke exceeded 30 mm, because the free end moved upwards during heating more than it moved downwards during RT bending.

These results prove that the specimens 3D printed from pure rPET and rPETG, as well as those printed from rPET/rPETG stratified composites, experienced free-recovery SME, after being RT-bend to 90° and heated to 100 °C. The different behavior of the rPET and rPETG layers, from the point of view of thermal expansion coefficient and elasticity modulus, might be the cause for the additional free-end’s displacement.

### 3.2. Evaluation of Glass Transition

**Figure 3:**
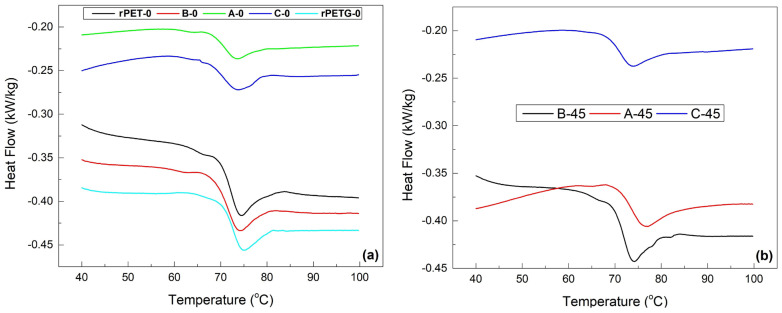
Variations of heat flow vs. temperature, of the rPET/rPETG composite specimen fragments, recorded by DSC, for the specimens with different raster orientations: (**a**) 0° and (**b**) 45°.

As previously discussed, the glass transition is considered the primary mechanism responsible for SME occurrence in polymers. During heating, the glass transition is associated with an endothermic step in heat flow variation with temperature, on the DSC thermographs, and with a marked decrease in the storage modulus, on the DMA plots [16]. These effects are emphasized in the following. The DSC thermograms recorded during the heating, from RT to 100 °C, of the rPET/rPETG composite fragments are summarized in Figure 3.

The endothermic steps, which are typically associated with glass transition, are followed by endothermic minima, which illustrate relaxation phenomena [22]. During the first heating, the endothermic minimum observed immediately after T_g_ corresponds to enthalpy relaxation (physical aging) of the amorphous phase. This peak can be characteristic of structural ageing by thermal relaxation at the glass transition. According to ISO 11357-2, glass transition temperatures must be evaluated from the heat flow variation step. On the other hand, the endothermic peak associated with relaxation must not be interpreted as melting or used for T_g_ determination [23]. In this case, only the endothermic slope was evaluated (T_onset_, T_mid_, T_inflection_, and T_end_), and the relaxation peak was not evaluated (relaxation peak temperature—T_relax_). The parameters of glass transition, determined by the software Proteus v.6.1, are summarized in Table 2. According to ISO 11357-2, the determination of the three characteristic glass transition temperatures is recommended as: T_onset_, T_infletion_, and T_end_. T_onset_ marks the beginning of the glass–rubber transition, T_infletion_ represents the temperature of maximum rate of change in heat capacity, and T_end_ indicates the completion of the transition. Evaluating all three temperatures is consequently fully compliant with international standards [23]. In this case, because rPET and rPEG exhibit broad glass transitions influenced by thermal history and relaxation effects, we intentionally reported all characteristic temperatures to fully describe the transition interval rather than relying on a single T_g_ value. The thermograms corresponding to specimens rPET-45 and rPETG-45 are similar to rPET-0 and rPETG-0, respectively. For these similarity and simplicity reasons, the DSC charts of these two specimens were not illustrated in Figure 3b.

The parameters comprise the temperatures for glass transition onset (T_onset_), middle range (T_mid_), inflection point (T_inflection_), and end (T_end_), as well as the variation of specific heat (ΔC_p_). These data show that all of the glass transitions were completed between 71 and 75.7 °C. However, according to Figure 2, the strokes were still developed during heating beyond these temperatures, up to 95 °C.

**Figure 4:**
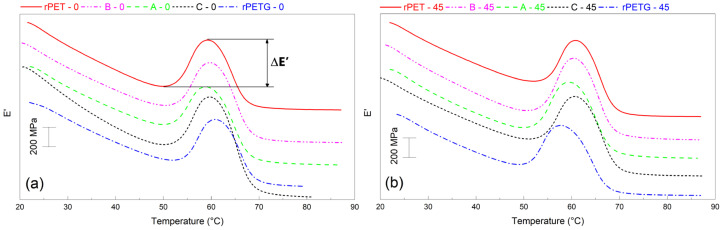
Variations of storage modulus vs. temperature, of the rPET/rPETG composite specimens with different raster orientations, recorded by DMA: (**a**) 0° and (**b**) 45°.

For a better insight into the change of mechanical properties of rPET/rPETG composite specimens during heating, the representative variations of storage modulus with temperature were illustrated in Figure 4.

As previously pointed out by some of the present authors, both rPET and rPETG experienced a sharp increase in the storage modulus during heating, before the glass transition thermal range [10,11]. In addition, it was pointed out that rPETG developed higher storage modulus maxima during both liquid nitrogen and air-cooling, as compared to those reached during heating [12]. On the other hand, it has been argued that the rPET specimens were first destabilized during heating, and this could be the cause of the storage modulus peak [24].

The local increase (ΔE’) and maximum values (E’_max_) of storage modulus and the corresponding temperature of the maxima are listed in Table 3.

The difference between T_g_ values determined by DSC and DMA is due to the different measuring principles of the two devices. The former measures heat flow variation, and the latter depends on heating and the sinusoidal stress applied, thus being more sensitive to the changes occurring within the polymer chains. Moreover, the two devices use different heating rates, namely 5 °C/min at DMA and 10 °C/min at DSC, which can cause the determination of different critical temperatures [25]. Among the data listed in Table 3, the highest storage modulus increase and maximum value were obtained for the specimen C-0, which developed the largest stroke in Figure 2a.

### 3.3. Evaluation of Tensile Behavior

The tensile behavior was evaluated by breaking three specimens of each type and by subjecting one specimen to a loading-unloading cycle of static tension. The values of the mechanical strength (F_max_—maximum force, E—Young modulus, σ_y 0.5_—yield stress at 0.5% strain, and σ_r_—failure stress) and plasticity (ε_y 0.5_—yield strain at 0.5% and ε_r_—failure strain) parameters for each of the tensile failure curves are listed in Table 4.

**Figure 5:**
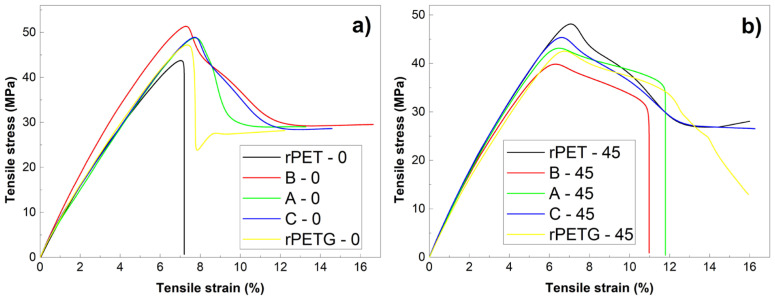
Representative tensile failure curves, determined as an average of three experimental curves for the specimens with different raster orientations: (**a**) 0° and (**b**) 45°.

According to ISO 527-2, Young’s modulus, E, was determined using the formula:

E = (σ_2_ − σ_1_)/(ε_2_ − ε_1_) (1)

where σ_1,2_ are the stresses corresponding to the strains ε_1_ = 0.0005 and ε_2_ = 0.0025, respectively. The yield stress, σ_y 0.5_, is considered as a function of the polymer’s type: fragile or ductile. For ductile polymers, σ_y 0.5_ is determined by the stress plateau. At fragile polymers, σ_y 0.5_ would correspond to a 0.5% strain [18]. σ_r_ and ε_r_ are the failure stress and strain, respectively. The representative tensile failure curves, determined as an average of three experimental curves, are illustrated in Figure 5.

When comparing the ten diagrams from Figure 5, it is rather obvious that the pure rPET and rPETG specimens behave differently, from the point of view of the influence of raster orientation, as compared to stratified composite rPET/rPETG specimens. Thus, the composite specimens 3D printed with 0° raster orientation, from Figure 5a, namely B-0, A-0, and C-0, experienced larger ultimate stresses (σ_r_) and strains (ε_r_) as compared to their counterparts, B-45, A-45, and C-45, respectively, printed with 45° raster orientation.

Moreover, the B-0, A-0, and C-0 specimens, as well as the pure rPETG-0 specimens, did not experience a clear failure tendency but rather developed stress plateaus, after a sharp necking, occurring around 8% strains. Conversely, at the specimens printed with 45° raster orientation, the necking occurred around 7% strains and was much smoother.

It is noticeable that, from the point of view of static tensile failure, no obvious variation tendency of mechanical parameters has been observed with varying the rPET/rPETG proportion.

**Figure 6:**
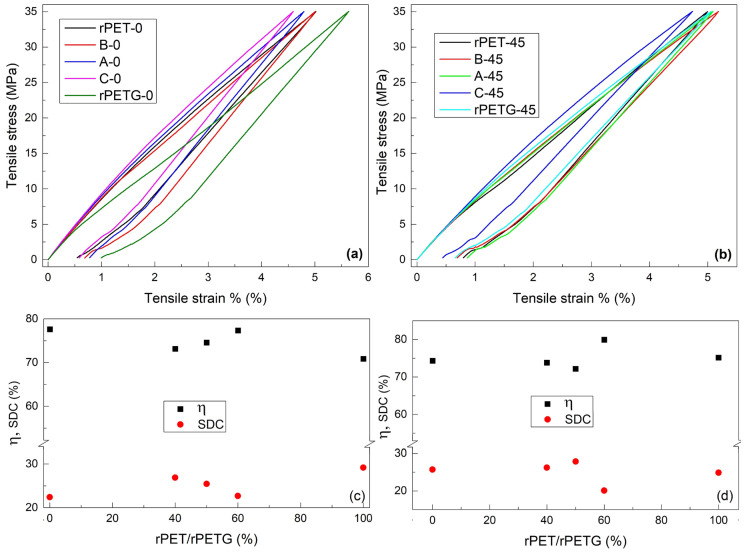
Representative tensile loading-unloading curves, and corresponding mechanical parameters for different raster orientations: (**a**) stress-strain curves at 0°; (**b**) stress-strain curves at 45°; (**c**) mechanical parameters at 0° raster and (**d**) mechanical parameters at 45°.

Considering that all ten average tensile failure curves did not show any breaking tendency for tensile stresses below 35 MPa, this stress was chosen as the maximum for tensile loading-unloading cycles. The results are summarized in Figure 6.

In this case, an obvious varying tendency can be observed at the maximum strain of 35 MPa. It decreased with increasing the rPETG fraction, in the order: 40% (B) → 50% (A) → 60% (C).

The mechanical parameters, illustrated in Figure 6, were defined as a function of the dissipated (E_1_) and unloading-released (E_2_) energies, proportional to the surface areas between loading–unloading curves and under the unloading curve, respectively [26]. Based on these energies, the energy storage efficiency (η) and specific damping capacity (SDC) were calculated with [27]:

η = E_2_/(E_1_ + E_2_) × 100 (2)

SDC = E_1_/(E_1_ + E_2_) × 100 (3)

Figure 6c illustrates a monotonic increasing tendency of energy storage efficiency, η, with increasing the amount of rPET/rPETG fraction. The typical values of energy storage efficiency, η, range between 70 and 80%, while those of specific damping capacity, SDC, are between 20 and 30%. It is noteworthy that the specimens C, which developed the largest free-recovery SME strokes and storage modulus increase, had the largest energy storage efficiency.

### 3.4. Evaluation of the Fractographic Morphology

**Figure 7:**
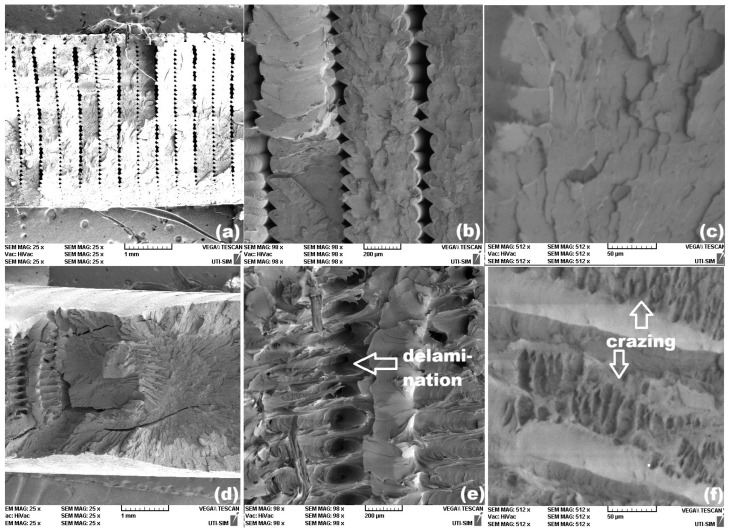
Representative SEM fractographs of rPET specimens with different raster orientations. 0°: (**a**) cross-section; (**b**) two-layer area and (**c**) detail of the fragile surface in one layer-area and 45°: (**d**) cross-section; (**e**) delamination area and (**f**) crazing area.

The SEM fractographs reveal marked differences between the cross-sections of the broken specimens, 3D printed with different raster angles, and no noticeable modifications between the similar specimens printed from pure rPET and rPETG. Figure 7 illustrates the representative morphological aspects of the broken surfaces of rPET specimens.

**Figure 8:**
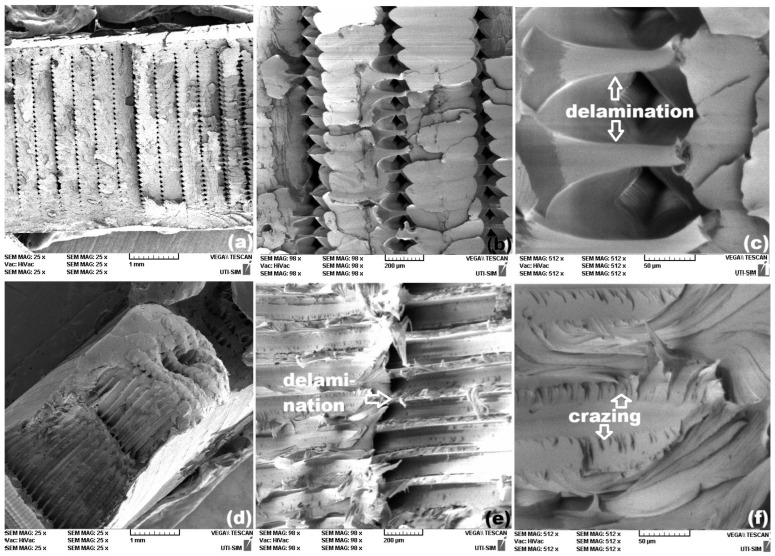
Representative SEM fractographs of rPETG-0 specimens with different raster orientations. 0°: (**a**) cross-section; (**b**) two-layer area and (**c**) detail of one layer-area with high-surface relief fragile region and a delamination zone, and 45°: (**d**) cross-section; (**e**) delamination area and (**f**) crazing area.

Similar aspects were noticeable for rPETG 3D printed specimens, with two different raster orientations, as illustrated in Figure 8.

The brittle character of the tension failure curves of specimens rPET-0 and r-PETG-0 is illustrated by sharp stress drops in Figure 5a. The SEM fractography from Figure 7c corresponds to a fragile failure of specimen rPET-0, due to the marked surface relief of the fractured surface, which is in agreement with the brittle character of failure [9].

On the other hand, the tensile failure curve of the specimen rPETG-0 shows both a sharp stress decrease and a stress plateau. The SEM fractography from Figure 8c reveals both high relief areas, caused by brittle failure, and delamination areas, which can be linked to the stress plateaus.

The micrographs of specimens rPET-45, from Figure 7e,f, and specimens rPETG-45, from Figure 8e,f, illustrate delamination and crazing areas. The former can be caused by the gradual tearing of many fibers out of the matrix [28], which can be associated with the stress plateaus [29] noticeable on the corresponding failure curves from Figure 5b. The latter appear as white, crack-like, sharply localized bands of plastically deformed material and can be associated with marked necking [30].

As compared to pure rPET or rPETG specimens, the fractographic features of the broken surfaces of 3D printed stratified composites with alternative rPET-rPETG layers experienced marked morphological changes.

Considering the printing scheme, shown in Figure 1, specimens B (with rPET/rPETG = 60/40) comprise an alternance of two rPET and one rPETG layers, which means that the rPET area has a double width as compared to the rPETG area. At specimen A, the alternance is one rPET and one rPETG layer since the composition is rPET/rPETG = 50/50.

**Figure 9:**
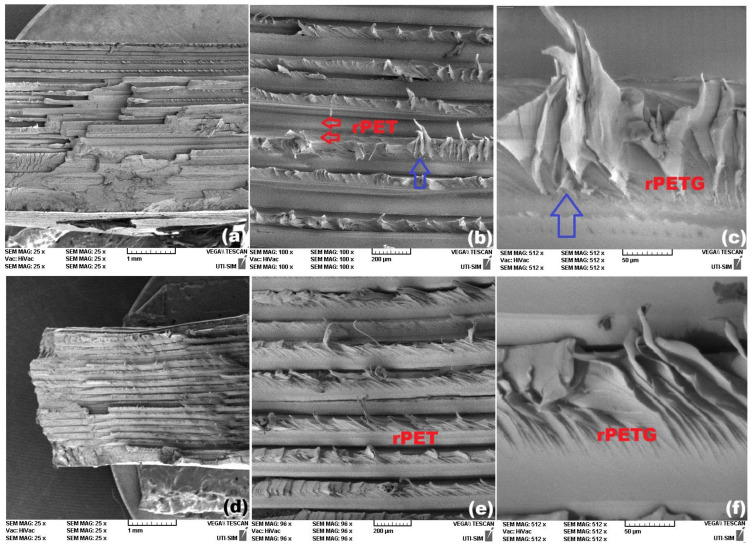
Representative SEM fractographs of the specimens 3D printed at 45° raster orientation, of stratified composites with two different rPET/rPETG fractions, namely B-45 (rPET/rPETG = 60/40): (**a**) cross-section; (**b**) illustration of the alternance of two rPET layers and one rPETG layer, and (**c**) detail of delamination area of the rPETG layer and A-45 (rPET/rPETG = 50/50): (**d**) cross-section; (**e**) delamination areas of rPETG layers, and (**f**) details of a delamination area.

During the static tensile failure, the rPET areas behave differently from the surrounding rPETG areas, considering that the former is softer than the latter [31]. The SEM fractographs from Figure 9 illustrate this aspect for both specimens with the compositions rPET/rPETG = 60/40 (B) and rPET/rPETG = 50/50 (A), which were 3D printed at 45° raster orientation.

At specimen B-45, the two rPET layers, being softer, could be continuously elongated during tensile testing, without any disruption. The single rPETG layer, being sandwiched between the soft rPET layers, could not accommodate their large deformations, failed, and developed high-relief scarps [32], such as those pointed out by blue vertical arrows in Figure 9b,c.

Similar aspects are noticeable at A-45 specimens, where the thicknesses of rPET and rPETG layers are equal. Due to the lower fraction of rPET layers, the rPETG layers were not subjected to as high strains as specimens B-45. Consequently, the scarp relief is lower in Figure 9f than in Figure 9c.

Aiming to relate the character of the tensile failure curves from Figure 5 with the morphological features of the SEM micrographs from Figure 7, Figure 8 and Figure 9, it can be assumed that the presence of the stress plateaus can be related to the presence of delamination areas, while the crazing areas can be associated with necking.

## 4. Conclusions

By summarizing the results presented and discussed in the previous section, the following conclusions can be drawn.

- From the point of view of free-recovery SME:
  ○ Most of the specimens printed at 0° developed larger strokes as compared to those printed at 45°;
  ○ The specimens C, with rPET/rPETG = 40:60, developed the largest strokes, exceeding 30 mm;
  ○ During shape recovery, some specimens deformed more than the RT straight shape and became concave.
- From the point of view of the properties change during the glass transition:
  ○ On the DSC charts, glass transition was completed between 71 and 75.7 °C;
  ○ The specimen C-0 printed at 0° experienced the highest storage modulus increase, 872 MPa, and maximum value, 1818 MPa.
- From the point of view of the tensile behavior:
  ○ The composite specimens, with different rPET/rPETG ratios, 3D printed at 0° developed larger ultimate stresses and strains than those printed at 45°;
  ○ Most of the specimens 3D printed at 0° developed stress failure plateaus;
  ○ The specimens 3D printed at 45° developed smoother necking;
  ○ The maximum strain reached at 35 MPa decreased with increasing the rPET/rPETG ratio, at stratified composite specimens;
  ○ At the specimens 3D printed at 0°, with increasing the rPET/rPETG ratio, storage modulus efficiency increased from 73 to 77% and specific damping capacity decreased from 27 to 23%;
  ○ The largest storage modulus efficiencies were obtained at the specimens that developed the largest SME strokes and storage modulus increases.
- From the point of view of fractographic morphology:
  ○ On the tensile failure curves, the presence of necking was associated with the formation of crazing areas and the failure stress plateaus with delamination zones;
  ○ At the specimens 3D printed at 45°, the rPETG layers failed before the rPET layers and developed a surface relief with scarps.

The unusual behavior of the 3D printed stratified composite lamellar specimens, which became concave during free-recovery SME development, can be associated with the different thermal expansion coefficients and stiffness of rPET and rPETG layers. A specific investigation on this phenomenon will make it the object of a subsequent study.

All the above features recommend the use of 3D printed rPET/rPETG stratified composites for the manufacture of executive elements of light actuators, operating at low temperatures.

## Figures and Tables

**Table 1:** Summary of 3D printing parameters.

	Parameter	Value		Parameter	Value
Quality	Layer height (mm)	0.1	Speed	First layer speed (mm/s)	80
	Initial layer height (mm)	0.1		Outer wall speed (mm/s)	100
	Outer wall width (mm)	0.45		Inner wall speed (mm/s)	100
	Inner wall width (mm)	0.45		Solid infill speed (mm/s)	150
	Solid fill width (mm)	0.45	Adhesion	Brim	Yes
	Internal solid width (mm)	0.45		Brim width (mm)	5
	Seam alignment	Aligned back		Brim-object gap (mm)	0.1
Strength	Wall loops	1	Material	Extrusion temperature (°C)	240
	Top shell layers	5 (DMA)		Build plate temperature (°C)	90
		20 (TENS)		Filament cooling fan speed	Min. (20%/12 s) Max. (50%/9 s)
	Top surface pattern	Monotonic line		z-hop height (mm)	0.4
	Bottom shell layers	5 (DMA)	Multimaterial	Purge block	Yes
		20 (TENS)		Puge block width (mm)	35
	Bottom surface pattern	Monotonic line		Purge block volume (mm^3^)	45
	Ensure vertical shell thickness	All		Minimal purge volume (mm^3^)	15

**Table 2:** Glass transition parameters corresponding to the DSC thermograms from Figure 3.

Specimen	T_onset_, °C	T_mid_, °C	T_inflection_, °C	T_end_, °C	ΔC_p_, J/kg°C
rPET-0	70	72	73	73	295
B-0	69	79	72	73	361
B45	70	72	72	72	189
A-0	68	71	71	72	169
A45	79	74	73	76	266
C-0	68	70	71	71	110
C-45	69	71	71	73	200
rPETG-0	71	73	73	74	288

**Table 3:** Values of temperature, local increase, and maximum of storage modulus, corresponding to the DMA thermograms from Figure 4.

Specimen	T, °C	ΔE’	E’_max_, MPa	Specimen	T, °C	ΔE’	E’_max_, MPa
rPET-0	59	820	1237	rPET-45	61	687	1258
B-0	60	760	1432	B45	61	870	1360
A-0	59	685	1402	A45	60	761	1265
C-0	60	872	1818	C-45	61	722	1323
rPETG-0	61	734	1217	rPETG-45	58	651	1145

**Table 4:** Mean and standard deviation values of the parameters of mechanical strength and plasticity, determined on the tensile failure curves.

Specimen	F_max_, N	E, MPa	σ_y 0.5_, MPa	ε_y 0.5_, %	σ_r_, MPa	ε_r_, %
rPET-0	1779 ± 32	885 ± 49	29 ± 1	0.04 ± 0.01	44 ± 1	7.09 ± 0.29
rPET-45	1928 ± 23	962 ± 40	29 ± 2	0.03 ± 0.00	48 ± 1	7.23 ± 0.03
B-0	2045 ± 14	1032 ± 7	26 ± 2	0.03 ± 0.00	50 ± 1	7.13 ± 0.20
B-45	1612 ± 26	924 ± 41	26 ± 3	0.03 ± 0.01	40 ± 1	7.55 ± 1.30
A-0	1931 ± 22	942 ± 85	21 ± 8	0.02 ± 0.01	48 ± 0.14	7.24 ± 0.74
A-45	1734 ± 18	957 ± 40	28 ± 3	0.04 ± 0.01	42 ± 0.33	7.38 ± 0.16
C-0	1967 ± 17	975 ± 17	21 ± 3	0.03 ± 0.01	49 ± 0.43	7.46 ± 0.11
C-45	1810 ± 8	968 ± 48	28 ± 2	0.03 ± 0.00	44 ± 0.21	7.16 ± 0.19
rPETG-0	1858 ± 39	939 ± 23	25 ± 3	0.03 ± 0.00	45 ± 2	6.80 ± 0.58
rPET-45	1704 ± 29	890 ± 34	25 ± 6	0.03 ± 0.01	41 ± 1	7.49 ± 0.11

## Data Availability

The original contributions presented in this study are included in the article/Supplementary Materials. Further inquiries can be directed to the corresponding author.

## Supplementary Materials

The following supporting information can be downloaded at: https://www.mdpi.com/article/10.3390/polym18030370/s1, Video S1. Free-recovery SME in the lamellar specimens of stratified composites.

## Abbreviations

The following abbreviations are used in this manuscript:

rPETG	Recycled polyethylene terephthalate glycol
PETG	Polyethylene terephthalate glycol
rPET	Recycled polyethylene terephthalate
TENS	Tension
PET	Polyethylene terephthalate
SME	Shape memory effect
SEM	Scanning electron microscopy
DSC	Differential scanning calorimetry
DMA	Dynamic mechanical analysis
SDC	Specific damping capacity
RT	Room temperature
